# Modification of the Tensile Performance of an Extruded ZK60 Magnesium Alloy with the Addition of Rare Earth Elements

**DOI:** 10.3390/ma16072828

**Published:** 2023-04-02

**Authors:** Soroush Najafi, Alireza Sheikhani, Mahdi Sabbaghian, Péter Nagy, Klaudia Fekete, Jenő Gubicza

**Affiliations:** 1 School of Metallurgy and Materials Engineering, College of Engineering, University of Tehran, Tehran P.O. Box 11155-4563, Iran; soroush.najafiran@gmail.com (S.N.); ali.ksa72@gmail.com (A.S.); msabbaghians@ut.ac.ir (M.S.); 2 Department of Materials Physics, Eötvös Loránd University, P.O. Box 32, H-1518 Budapest, Hungary; nagyp@student.elte.hu; 3 Department of Physics of Materials, Faculty of Mathematics and Physics, Charles University, 121 16 Prague, Czech Republic; fekete@karlov.mff.cuni.cz

**Keywords:** ZK60 alloy, rare earth element, dislocation density, texture, work hardening

## Abstract

The influence of rare earth (RE) elements on the microstructure and mechanical performance of an extruded ZK60 Mg alloy was studied. Two types of RE elements were added to a ZK60 material and then extruded at a ratio of 18:1. The first new alloy contained 2 wt% Y while the second one was produced using 2 wt% Ce-rich mischmetal. The microstructure, the texture, and the dislocation density in a base ZK60 alloy and two materials with RE additives were studied by scanning electron microscopy, electron backscattered diffraction, and X-ray line profile analysis, respectively. It was found that the addition of RE elements caused a finer grain size, the formation of new precipitates, and changes in the initial fiber texture. As a consequence, Y and Ce-rich RE elements increased the strength and reduced the ductility. The addition of these two types of RE elements to the ZK60 alloy decreased the work hardening capacity and the hardening exponent mainly due to grain refinement.

## 1. Introduction

Magnesium (Mg) and its alloys are promising materials for automobile or aerospace applications or also as biodegradable implants. This extensive capability is due to properties such as low density, high specific strength, good castability, a controllable corrosion rate, and biocompatibility [1,2]. However, there are poor mechanical features of Mg, e.g., the low room temperature formability [2,3]. For obtaining the better mechanical behavior of Mg alloys, various strategies have been developed, such as hot deformation, dynamic recrystallization (DRX), and alloying with additional elements [3,4,5]. This study investigates the latter strategy in a ZK60 Mg alloy.

The ZK60 alloy is one of the most common Mg alloys with acceptable strength and ductility, in which the main alloying elements are Zn and Zr in concentrations of about 6 and 0.5 wt%, respectively. Hot extrusion of a ZK60 alloy helps to improve the strength due to grain refinement [4,6]. In hot deformation processes such as extrusion, DRX at grain boundaries and the formation of twins cause grain refinement [4,7]. Other methods such as severe plastic deformation (SPD) techniques can also refine the grain structure of a ZK60 alloy. For instance, Fakhar and Sabbaghian reported that applying five passes of repeated upsetting (RU), as a powerful SPD method, could reduce the grain size of a rolled and annealed ZK60 alloy from 40 μm to 2.8 μm [8].

Alloying is also an effective way to medicate the mechanical performance of Mg alloys. It has been reported that the addition of rare earth (RE) elements can improve the mechanical properties at ambient and high temperatures through the formation of thermally stable precipitates with high melting points at the grain boundaries and inside the grains [6,9,10]. RE elements also influence mechanical properties by changing the crystallographic texture. Due to the phenomenon of particle-stimulated nucleation (PSN), precipitates also influence grain size, especially if they have a high volume fraction [11]. Owing to the hexagonal close-packed (HCP) structure of Mg alloys, their mechanical behavior also depends on the activation of different dislocation slip systems, which is influenced by their texture [12,13,14]. Li et al. proposed that a Yb addition effectively refined recrystallized grains and yielded dense Mg–Zn–Yb nanoprecipitates that could inhibit grain boundary migration, and also could promote the formation of the RE texture component [15]. It was reported that the yield stress of the ZK60 alloy increased from 212 MPa to 308 MPa in a ZK60–3Ce alloy due to the development of a finer grain structure and a large volume fraction of secondary phase particles (MgZn_2_Ce) [6].

The improvement of work hardening (WH) behavior is one of the most important ways to enhance both the strength and workability of Mg alloys [16]. The enhanced hardening capability leads to resistance against the creation of tensile mechanical instabilities and therefore improves ductility. It has been shown that RE elements have a significant effect on the strength and WH behavior of Mg alloys [17]. In our previous research, we studied the effects of 1 and 2 wt% RE elements on the shear strength of a ZK60 alloy extruded at a ratio of 12:1 [18]. It was reported by Zhou et al. that the WH in an extruded Mg–2Nd–0.5Zr alloy was low, most probably due to the high density of basal dislocations and the relatively low density of non-basal dislocations during deformation [19]. Shi et al. found that an improved WH capability was achieved for an extruded Mg-6Zn-1Gd-0.3Ca alloy with an increased volume fraction of recrystallized grains, which was attributed to the higher dislocation storage capacity in the recrystallized volumes [20]. In that study, the volume fractions of the recrystallized and the coarse deformed grains were tailored by rolling and subsequent annealing under different conditions. It was also observed that when the ratio of the volumes of recrystallized grains and coarse deformed grains increased, the WH rate decreased in stage Ⅲ, while an opposite trend was detected in stage Ⅳ for a Mg–6Zn-1Gd-0.12Y alloy. These effects were closely related to the initial dislocation density in the rolled and annealed samples, as well as the dislocation evolution under tension [21].

In this study, the change in the tensile performance of an extruded ZK60 alloy (at an extrusion ratio of 18:1) due to the addition of RE elements is investigated, and its mechanical behavior (strength and WH) is related to its microstructure and crystallographic texture. In addition to the base ZK60 alloy, two other compositions were processed. One sample contained 2 wt% Y while to the other alloy a Ce-rich mixture of large RE elements of a total fraction of 2 wt% was added. This mixture of RE alloying elements contained Ce, La, Nd, and Pr. The dislocation density in the extruded samples with or without RE addition was studied by X-ray line profile analysis (XLPA). The grain structure and the crystallographic texture were analyzed by electron back-scattered diffraction (EBSD). The effect of the addition of RE elements on the microstructure and the mechanical performance of the ZK60 alloy was discussed. To the knowledge of the authors, such a careful study of the WH behavior of ZK60 alloys containing RE elements is missing from the literature.

## 2. Materials and Methods

Three alloys were investigated in this study: a ZK60 Mg-based alloy with the alloying element concentrations of 6 wt% Zn and 0.5 wt%, Zr, and two other materials in which 2 wt% Y or 2 wt% Ce-rich RE elements were added to the ZK60 alloy (the relative fractions of the constituents in wt% were 50.6% Ce, 23.2% La, 20.3% Nd, and 5.9% Pr). Similar to our previous work [18], all three materials were prepared by melting them in an induction furnace at 740 °C. After casting and homogenization at 440 °C for 12 h, extrusion was performed on the samples at 180 °C at the extrusion ratio of 18:1.

The phase composition of the alloys was analyzed by X-ray diffraction (XRD) using a Smartlab diffractometer (manufacturer: Rigaku, Tokyo, Japan). In the XRD experiments, a divergent beam was applied in Bragg–Brenatno (*θ*–*θ*) diffraction geometry using CuKα radiation (wavelength: 0.15418 nm).

The microstructure was studied by scanning electron microscopy (SEM) using a ZEISS SIGMA VP microscope (manufacturer: Carl Zeiss AG, Jena, Germany). During the surface preparation, the samples were polished with an alumina suspension (particle size: 0.5 μm) and then etched in a picral solution at room temperature. The same instrument was used for energy-dispersive X-ray spectroscopy (EDS) in order to determine the chemical composition of the secondary phases.

The crystallite size and the dislocation density were investigated by XLPA [22]. This analysis was carried out on diffractograms measured by a high-resolution rotating anode diffractometer (type: RA-MultiMax9, manufacturer: Rigaku, Tokyo, Japan) using CuKα_1_ radiation (wavelength: 0.15406 nm). The measured XRD patterns were evaluated by the convolutional multiple whole profile (CMWP) fitting method which yielded the crystallite size and the dislocation density in the studied samples. In this procedure, all peaks in the measured pattern are fitted simultaneously using theoretical profile functions calculated on the basis of the kinematical theory of XRD [23]. These calculated functions contained unknown parameters of the microstructure, such as the median and the variance of the crystallite size distribution and the dislocation density, which were then changed during the CMWP procedure in order to obtain the best fit between the measured and the calculated patterns. It should be noted that the calculated XRD pattern contains not only the diffraction peaks but also the background under the peaks which was approximated by a spline. The strongest 16 reflections of the main Mg phase in the diffraction angle (2*θ*) range between 30 and 125° were used in the evaluation. An example for CMWP fitting will be shown in Section 3.1. It should be noted that the recent development in XLPA enables the determination of the microstructural parameters with the help of artificial intelligence [24]. This novel machine learning-based method is much easier to use and faster than the conventional pattern-fitting procedure. However, the present Mg alloys cannot be evaluated by this new method since it is available only for face-centered cubic (fcc) structures as yet.

The EBSD technique was used to investigate the crystallographic texture and to characterize the grain boundaries in the samples. First, the surface of the specimens was mechanically polished with a diamond paste down to 1 µm, and then refined by ion beam polishing using a Leica EM RES102 system (manufacturer: Leica Mikrosysteme, Wetzlar, Germany). The EBSD was carried out with Zeiss Cross Beam Auriga SEM (manufacturer: Carl Zeiss AG, Jena, Germany) with a step size of 0.8 μm. The misorientation distribution was determined by the EDAX OIM software (manufacturer: EDAX Inc., Mahwah, NJ, USA). In addition, the OIM software was also used for the determination of the area-weighted mean grain size from the EBSD images. In this evaluation, the regions containing at least 4 pixels and bounded by high-angle grain boundaries (HAGBs) with misorientation angles higher than 15° were considered grains.

An electrodischarge machine (EDM) was used to prepare tensile specimens parallel to the extrusion direction (ED) according to the ASTM E8-04 standard. The tensile tests were carried out on at least three samples for each condition using a SANTAM tensile machine with a capacity of 2 tons and at an initial strain rate of 10^–3^ s^–1^. The samples containing Y and the specimens with Ce-rich rare earth elements are denoted as ZK60–2Y and ZK60–2RE, respectively.

## 3. Results and Discussion

### 3.1. Microstructure and Texture of the Extruded ZK60 Alloys with and without RE Addition

**Figure 1:**
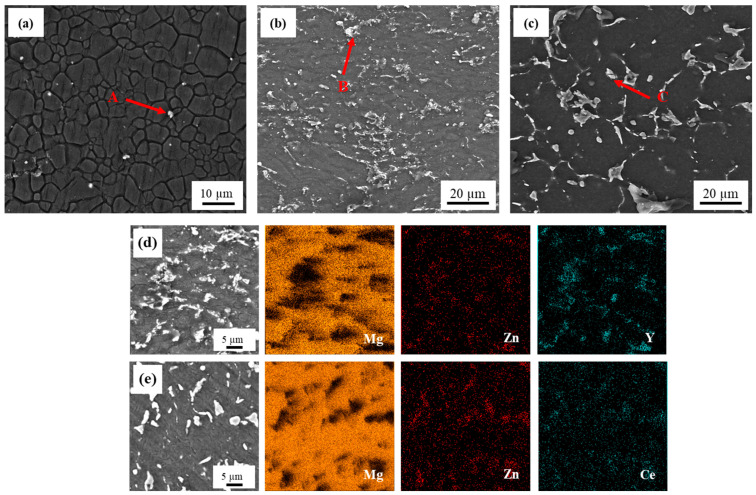
SEM images for (**a**) ZK60, (**b**) ZK60–2Y, and (**c**) ZK60–2RE alloys. The bright areas in the SEM images correspond to the secondary phase particles. The red arrows and the letters A, B and C show the locations where the EDS analysis was performed. The corresponding EDS spectra are shown in Figure 2. Color-coded EDS elemental maps for (**d**) ZK60–2Y and (**e**) ZK60–2RE alloys (Mg: orange, Zn: red, Y and Ce: turquoise).

**Figure 2:**
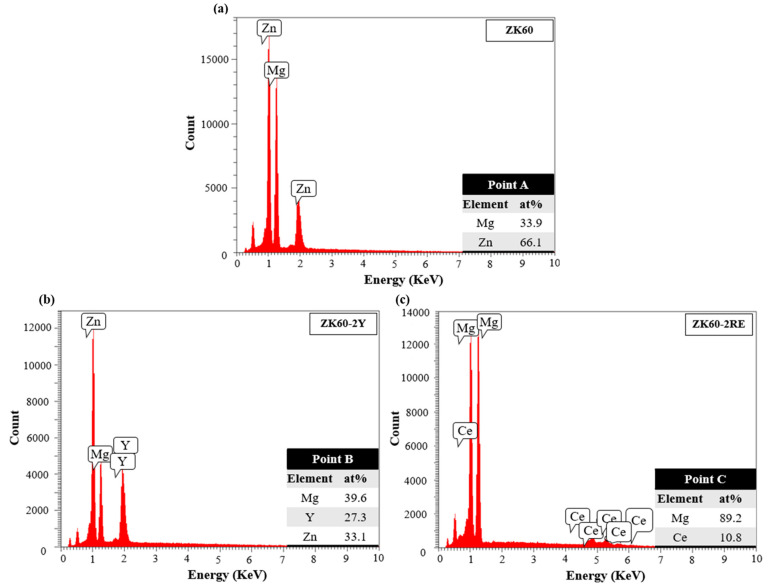
EDS spectra corresponding to (**a**) point A in Figure 1a, (**b**) point B in Figure 1b, and (**c**) point C in Figure 1c.

The SEM micrographs in Figure 1a–c revealed that the addition of Y and Ce-rich elements to the ZK60 alloy increased the volume fraction of precipitates from 2% to 14% and 15% in the ZK60–2Y and ZK60–2RE alloys, respectively (see also Table 1). Spherical and network-like precipitates were observed in both the Y- and RE-containing alloys; however, the ZK60–2RE alloy contained more network-like secondary phase particles than the ZK60–2Y alloy did. Figure 1d,e shows color-coded EDS elemental maps for the ZK60–2Y and ZK60–2RE alloys (Mg: orange, Zn: red, Y and Ce: turquoise). The EDS analysis suggests that the precipitates were enriched in Y and Zn in the ZK60–2Y alloy, and in Ce and Zn in the ZK60–2RE sample. Namely, the secondary phase particles indicated by the red arrows in Figure 1a–c had the following composition, as obtained by EDS (in at.%): 66.1% Mg and 33.9% Zn for the ZK60 alloy, 29.6% Mg, 33.1% Zn and 27.3% Y for sample ZK60–2Y, and 89.2% Mg and 10.8% Ce for the ZK60–2RE alloy. The corresponding EDS spectra are shown in Figure 2.

**Figure 3:**
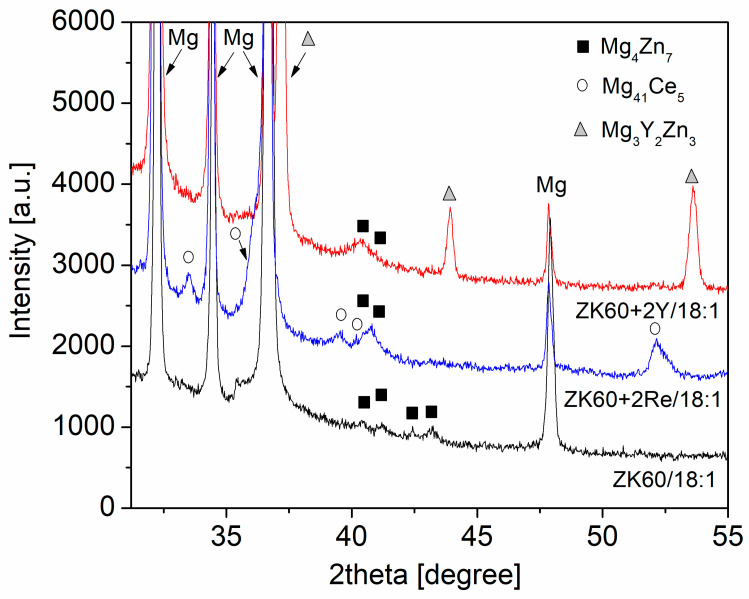
A part of the XRD patterns for the studied alloys.

The structure of the secondary phase particles was studied by XRD. A part of the XRD patterns containing peaks of the precipitates is shown in Figure 3. Using the ICDD PDF-2018 database, the particle structures were identified. It was found that the particles in the ZK60 alloy were Mg_4_Zn_7_ (PDF card no.: 01-071-9625) with a monoclinic structure. The magnitude of the edge vectors of the cell (i.e., the lattice constants) are a = 2.596 nm, b = 0.524 nm, and c = 1.428 nm. The angles between these edge vectors are α = 90°, β = 102.5°, and γ = 90°. In the ZK60 alloy, the precipitates were identified as Mg_3_Y_2_Zn_3_ (PDF card no.: 00-036-1275) with an fcc structure (the lattice constant is 0.6833 nm). In the ZK60–2RE alloy, the secondary phase particles were identified as Mg_41_Ce_5_ (PDF card no.: 01-071-7012) with a tetragonal crystal structure. The lattice constants of this structure are a = 1.478 nm and c = 1.043 nm. It is worth noting that there is reasonable agreement between the chemical compositions determined by EDS (see the former paragraph) and the nominal compositions of the precipitate structures identified by XRD. The slight difference could have been caused by the fact that it was not possible to exclude a fraction of the EDS signal that came from the material beneath the particles. In addition, the actual compositions of the precipitates may have slightly differed from the nominal chemical compositions given in the XRD cards without changing the structure; therefore, these small differences could not be observed by XRD. Similarly to SEM, XRD also suggested a higher amount of precipitates in the ZK60–2Y and ZK60–2RE alloys compared to the ZK60 alloy (see Figure 3).

**Figure 4:**
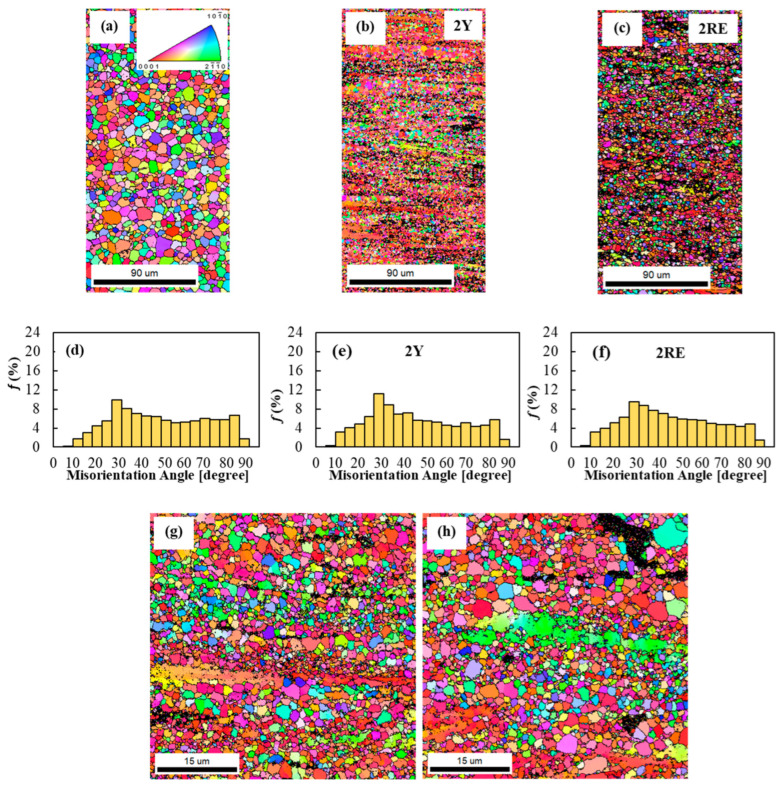
(**a**–**c**) EBSD grain orientation maps (the extrusion direction is horizontal), and (**d**–**f**) misorientation angle distribution histograms for ZK60, ZK60–2Y, and ZK60–2RE alloys, and higher magnification EBSD maps for (**g**) ZK60–2Y, and (**h**) ZK60–2RE alloys.

**Figure 5:**
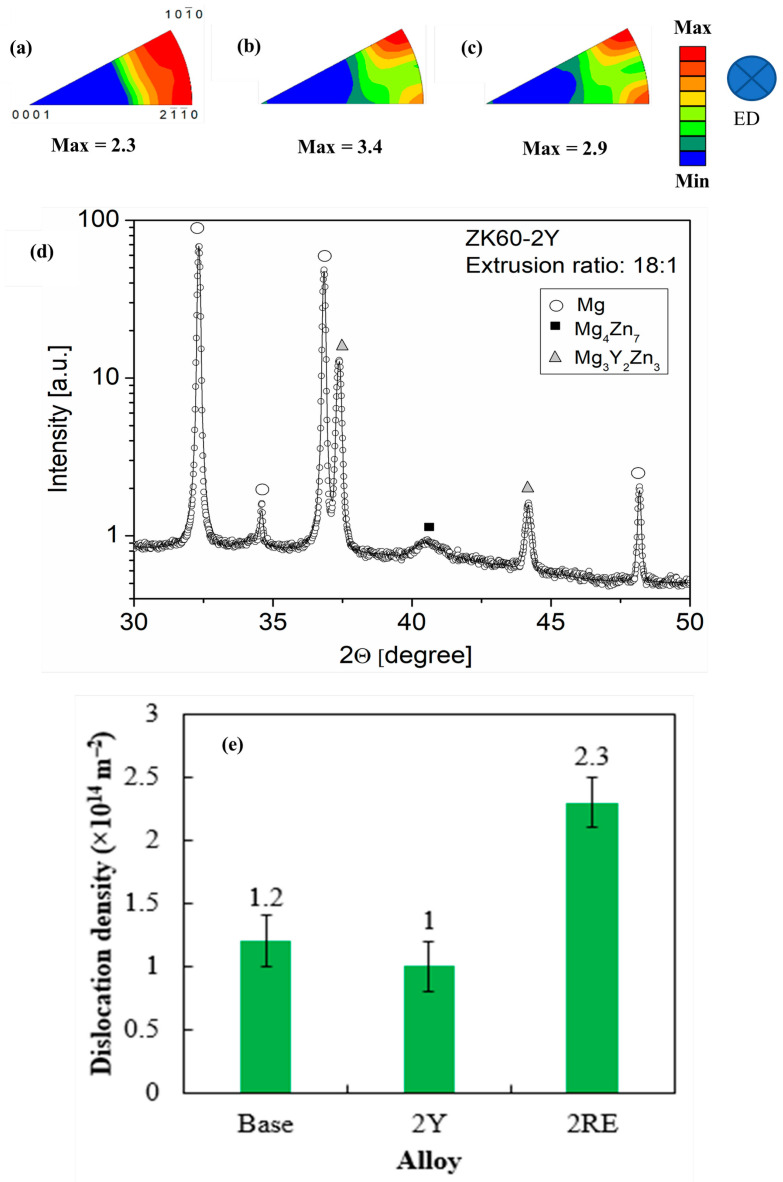
Inverse pole figures for (**a**) ZK60, (**b**) ZK60–2Y, and (**c**) ZK60–2RE alloys, and (**d**) CMWP fitting for the extruded ZK60–2Y alloy. The open circle and the solid line represent the measured data and the fitted XRD pattern, respectively. The intensity is in a logarithmic scale. (**e**) The dislocation density for the three studied alloys determined by CMWP fitting.

The grain orientation maps and misorientation angle distribution histograms obtained by EBSD for the three alloys are shown in Figure 4. The analysis of the EBSD images revealed that the grain size of the ZK60 alloy was reduced from 6.5 μm to 2.1 μm and 2.8 μm when Y and Ce-rich RE elements were added to the base alloy, respectively (Figure 4a–c). According to the misorientation angle distribution histograms (see Figure 4d–f), the fraction of HAGBs (*θ* > 15˚) was more than 90% for all three alloys. In addition, the presence of a sharp peak at 30˚ indicated the occurrence of continuous DRX (CDRX) [25]. Thus, it is suggested that the fine grains in the extruded samples formed due to recrystallization. On the other hand, some large elongated grains were present in the ZK60–2Y and ZK60–2RE alloys (see Figure 4g,h). These grains can be attributed to the segregation of large atoms, such as Ce, La, Nd, and Pr or Y, due to dislocations and grain boundaries which hindered recrystallization during extrusion and increased the required strain to complete the DRX process [18]. On the other hand, coarse secondary phase particles (usually larger than 1 μm) could promote PSN as a DRX mechanism in the ZK60–2Y and ZK60–2RE alloys [26]. It is obvious from Figure 5a that the predominant texture component for all alloys was fiber-like, in which the c-axis of the HCP crystals were aligned perpendicular to ED, and the (10$\bar{1}$0) and (2$\bar{1}\bar{1}$0) planes were parallel to the surface analyzed by EBSD. This type of texture is common in extruded Mg alloys and limits their formability due to the limited activation of easy glide systems [2]. It is evident in Figure 5a–c that the texture intensity increased with the addition of Y and Ce-rich RE elements. This type of texture change can be explained by the higher volume fractions of un-DRXed grains (see Figure 4a–c).

The average crystallite size and the dislocation density were determined by the XLPA method. As an example, Figure 5d demonstrates the CMWP fitting on the X-ray diffraction pattern obtained for the ZK60–2Y alloy. Only a part of the fitted pattern is shown in the figure. Table 1 shows that the crystallite size decreased with the addition of RE alloying elements. This trend is in line with the grain size reduction observed by EBSD. On the other hand, the crystallite size obtained by XLPA was much smaller than the grain size determined by EBSD. This phenomenon is well-known in plastically deformed metallic materials and is caused by the hierarchical nature of the deformed microstructures [22]. Indeed, during deformation, dislocations are formed and arranged into low-angle grain boundaries and/or dipolar walls in order to reduce the energy of the dislocation structure. The volumes separated by these dislocation boundaries exhibit low misorientations which break the coherency of the scattered X-rays; therefore, XLPA detected these volumes as individual crystallites which were considerably smaller than the grain size.

XLPA revealed that in the extruded ZK60 alloy the dislocation density was about 1.2 × 10^14^ m^−2^, which did not change remarkably due to the addition of 2% Y (see Figure 5e). On the other hand, the dislocation density increased to 2.3 × 10^14^ m^−2^ in the ZK60–2RE alloy. The higher dislocation density for sample ZK60–2RE can be attributed to the alloying effect since the large RE atoms could hinder the dislocation annihilation during the extrusion either by dissolving into the Mg matrix or by forming precipitates. It is surprising that the addition of 2% Y did not yield an increased dislocation density in the extruded sample. This effect can be understood if we assume that most yttrium was concentrated in the precipitates. Since a major fraction of these particles formed at the grain boundaries, as suggested by SEM (see Figure 1), they had no significant effect on the dislocation multiplication and annihilation occurring in the grain interiors. Therefore, the yttrium addition did not increase the dislocation density compared to the base ZK60 alloy. For sample ZK60–2RE, the relative fraction of RE atoms in the precipitates was much lower than that for specimen ZK60–2Y. Namely, the fractions of RE atoms in Mg_41_Ce_5_ and Mg_3_Y_2_Zn_3_ particles were 11% and 25%, respectively. On the other hand, the volume fractions of precipitates in the two alloys were similar (see Table 1). Therefore, most probably, the ZK60–2RE alloy contained more solute RE atoms in the grain interiors than the ZK60-2Y alloy did, which possibly contributed to the higher dislocation density after extrusion. In addition, the average size of the Ce, La, Nd, and Pr atoms present in the ZK60-2RE sample was about 5% larger than that of the Y added to the ZK60-2Y alloy. Therefore, the pinning effect of the former elements should have been higher on dislocations compared to that of the latter one. This effect coud have also contributed to the higher dislocation density in sample ZK60-2RE.

### 3.2. Influence of RE Element Addition on the Mechanical Properties of the Extruded ZK60 Alloy

**Figure 6:**
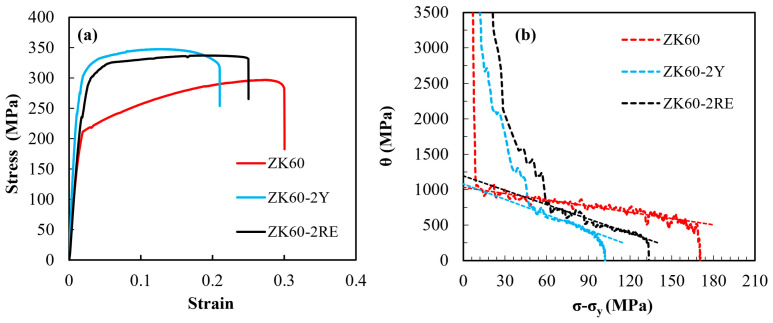
(**a**) Tensile engineering stress–strain curves and (**b**) work hardening curves. The lines fitted on the hardening curve in stage III were used for the determination of the values of $\theta_{0}^{III}$ and *σ*_s_ for the three studied alloys.

Figure 6a shows the tensile engineering stress–strain curves for all three alloys. The ultimate tensile strength (UTS) values of the ZK60, ZK60–2Y, and ZK60–2RE alloys were 299, 348, and 337 MPa, while the elongation to failure values were about 30, 21, and 25%, respectively. The formation of the new precipitates and their larger volume fraction due to the addition of Y or Ce-rich elements increased the strength of the two new alloys compared to the ZK60 counterpart. Similar results have been reported for other Mg alloys containing RE elements. For example, according to the research carried out by Sabbaghian et al., the UTS of an extruded Mg–4Zn alloy was enhanced from 301 to 336 MPa after the addition of 1 wt% Gd due to grain boundary hardening, particle strengthening, and texture hardening [27]. Moreover, another study revealed that the UTS of an extruded ZK60 alloy increased from 336 to 378 MPa when 2 wt% Yb was added [15]. In the present case, the addition of Y and Ce-rich RE alloying elements had different strengthening effects. The volume fractions of the secondary phase particles in the ZK60-2Y and the ZK60-2RE alloys were comparable; therefore, their hardening effects could be expected to be similar. On the other hand, the grain size hardening should have been higher for the ZK60–2Y alloy due to its smaller grain size (see Table 1), in accordance with the Hall–Petch relationship [28]. In addition, the strengthening contribution of the crystallographic texture was also higher for sample ZK60-2Y due to the enhanced texture intensity (Figure 5). At the same time, the dislocation density was much lower for ZK60-2Y alloy compared to ZK60-2RE which yielded a reduced dislocation strengthening contribution in the former material. However, the lower dislocation hardening was overwhelmed by the higher grain size and texture strengthening effects, resulting in a higher strength for the ZK60-2Y alloy than for the ZK60-2RE material.

Regarding the ductility, the reduced elongation to failure values for ZK60–2Y and ZK60–2RE alloys compared to the initial alloy was basically caused by the reduced grain size, since for smaller grains, the saturation of the dislocation density usually occurs earlier [22,29]. In addition, an increased amount of precipitates at grain boundaries can cause easier crack nucleation and propagation since these particles may act as stress concentration sites under loading [25,27]. The significance of the weakening effect of precipitates on grain boundary strength was more pronounced in the present ZK60–2Y and ZK60–2RE samples, in which, due to the small grain size, the applied loads were higher than that for the ZK60 alloy. Consequently, the base ZK60 alloy exhibited a greater elongation to failure than the new alloys with RE additives did.

For investigating the WH behavior, the WH rate (*θ*) was determined according to the relationship:

(1) $$\theta =\frac{d\sigma }{d\varepsilon },$$

where *σ* is the true stress and *ε* is the true strain. Figure 6b shows the *θ* versus *σ* − *σ_y_* curve for the three studied alloys where *σ_y_* is the yield strength. The hardening capacity was characterized by the following equation [30]:

(2) $$H_{c}=\frac{\sigma_{UTS}-\sigma_{y}}{\sigma_{y}}.$$

In Figure 6b, the approximately linear part of the curves is related to stage III which is usually described with the help of the Voce equation, expressed as:

(3) $$\theta =\theta_{0}^{III}(1-\frac{\sigma }{\sigma_{s}}),$$

where *σ*_s_ is the saturation stress. The values of $\theta_{0}^{III}$ and *σ*_s_ were obtained from the slope and the intercept of the straight line fitted to the stage III segment of the *θ* versus *σ* − *σ_y_* curve in Figure 6b. For the comparison of the WH behavior of the three alloys, the WH parameters are listed in Table 2. With the addition of 2% RE elements, the $\theta_{0}^{III}$ of the ZK60 alloy increased from about 1016 MPa to the range between 1090 and 1194 MPa, which was due to the stronger initial texture. The hardening capacity (*H*_c_) for the ZK60 alloy was 0.81, which decreased to 0.34 and 0.47 for the ZK60–2Y and ZK60–2RE alloys, respectively. The change in *σ*_s_ was similar to *H*_c_, and with the addition of RE, the value of *σ*_s_ reduced. Grain refinement in the ZK60–2RE alloy and especially in the ZK60–2Y alloy reduced the WH parameters. A similar result was obtained in a former study in which it was also suggested that the increase in the Mn content from 0 to 1.88 wt% in an extruded Mg–1Sn alloy decreased the *H*_c_, which was attributed to grain refinement [31]. In addition, the uniform plastic deformation stage in the tensile curve was fitted by a power-law constitutive equation:

(4) $$\sigma =K\varepsilon^{n},$$

where *K* is the strength coefficient, and *n* is the WH exponent. A higher value of *n* indicates a more pronounced WH. It can be seen in Table 2, that *n* is influenced by the addition of RE, and its value decreased from 0.3 to 0.13 and 0.19 for the ZK60–2Y and ZK60–2RE alloys, respectively. The reduction of *n* can be related to the decrease in ductility of RE-containing alloys, as shown in Figure 6a. The lower *n* value for the RE-containing alloys suggests an early saturation of hardening with the increasing strain which is a typical feature of metallic materials with reduced grain size. Indeed, the addition of RE elements resulted in a significant decrease in grain size as shown in Table 1. Additionally, with the addition of RE, the probability of activation of nonbasal slip systems increased due to the higher stress level in the samples during straining (see Figure 6a) [32]. All of these factors caused the reduction in the WH parameters of the RE-containing alloys compared to the ZK60 alloy. For the ZK60–2Y alloy, which had the smallest grain size, the values of the WH parameters are the lowest among the three studied samples.

## 4. Conclusions

The influence of the addition of 2 wt% Y and Ce-rich RE elements on the microstructure and mechanical behavior of an extruded ZK60 alloy was investigated. The following conclusions were drawn from the experimental results:

(1) Adding RE elements to the ZK60 alloy caused grain refinement, the formation of new precipitates, and an enhanced volume fraction of precipitates. The grain size reduction from 6.5 μm to 2.1 and 2.8 μm due to the addition of Y and Ce-rich RE elements, respectively, was attributed to the nucleation of new grains due to CDRX and PSN phenomena, and the pinning effects of second phase particles that hindered grain growth.
(2) In the RE-containing alloys, the texture intensity increased, and the highest texture intensity was achieved in the ZK60–2Y alloy. The higher texture intensity was due to the higher volume fractions of un-DRXed grains in the materials alloyed with Y and Ce-rich RE elements. The higher dislocation density in the ZK60-2RE alloy compared to the ZK60-2Y material can be explained by the expected higher concentration of solute atoms and their larger atomic radii compared to sample ZK60-2Y.
(3) The addition of RE elements increased the strength due to the solute and precipitate strengthening, and the grain size hardening. The ZK60–2Y and ZK60–2RE alloys exhibited lower ductility than the base alloy did due to the smaller grain size and the weakening effect of secondary phase particles on the grain boundary strength. These precipitates acted as stress concentration sites under loading, resulting in crack nucleation and/or easier crack propagation along the grain boundaries. The cracking was also facilitated by the higher stress level caused by the small grain size. The ZK60–2Y alloy exhibited the lowest work hardening rate due to the smallest grain size.

## Figures and Tables

**Table 1:** Microstructural characteristics and mechanical properties of the three studied alloys (*f*_P_: fraction of secondary phase particles as obtained by SEM; *f*_DRX_: fraction of recrystallized volumes determined by EBSD; *f*_HAGB_: HAGB fraction obtained by EBSD; *ρ*: dislocation density determined by XLPA; UTS: ultimate tensile strength).

Alloy	Grain Size (µm)	*f*_P_ (%)	*f*_DRX_ (%)	*f*_HAGB_ (%)	*ρ* (10^14^ m^−2^)	Crystallite Size (nm)	σ_y_ (MPa)	UTS (MPa)
ZK60	6.5 ± 0.3	2 ± 0.5	96.1	95.2	1.2 ± 0.2	119 ± 14	212 ± 9	299 ±11
ZK60–2Y	2.1 ± 0.2	14 ± 1	63.4	92.5	1.0 ± 0.2	96 ± 10	303 ± 6	348 ± 8
ZK60–2RE	2.8 ± 0.1	15 ± 1	61.2	92.7	2.3 ± 0.2	74 ± 10	299 ± 7	337 ± 5

**Table 2:** Extrapolated WH limit in stage III ($\theta_{0}^{III}$), corresponding saturation stress (σs), hardening capacity (Hc), and work hardening exponent (n) for the three studied alloys.

Alloy	$\theta_{0}^{III}$ (MPa)	*σ*_s_ (MPa)	*H*_c_	*n*
ZK60	1016	170	0.81	0.30
ZK60–2Y	1090	102	0.34	0.13
ZK60–2RE	1194	134	0.47	0.19

## Data Availability

The raw/processed data required to reproduce these findings cannot be shared at this time as the data also form part of an ongoing study.

## References

[1] Tsakiris V., Tardei C., Clicinschi F.M. (2021). Biodegradable Mg Alloys for Orthopedic Implants—A Review. J. Magnes. Alloys.

[2] Sabbaghian M., Mahmudi R., Shin K.S. (2020). Microstructure, Texture, Mechanical Properties and Biodegradability of Extruded Mg–4Zn‒xMn Alloys. Mater. Sci. Eng. A.

[3] Yu J., Zhang Z., Wang Q., Yin X., Cui J., Qi H. (2017). Dynamic Recrystallization Behavior of Magnesium Alloys with LPSO during Hot Deformation. J. Alloys Compd..

[4] Dong S., Jiang Y., Dong J., Wang F., Ding W. (2014). Cyclic Deformation and Fatigue of Extruded ZK60 Magnesium Alloy with Aging Effects. Mater. Sci. Eng. A.

[5] Gu D., Peng J., Wang J., Pan F. (2021). Effect of Mn Modification on Microstructure and Mechanical Properties of Magnesium Alloy with Low Gd Content. Met. Mater. Int..

[6] Banijamali S.M., Palizdar Y., Najafi S., Sheikhani A., Soltan Ali Nezhad M., Valizadeh Moghaddam P., Torkamani H. (2021). Effect of Ce Addition on the Tribological Behavior of ZK60 Mg-Alloy. Met. Mater. Int..

[7] Pourbahari B., Mirzadeh H., Emamy M., Roumina R. (2018). Enhanced Ductility of a Fine-grained Mg–Gd–Al–Zn Magnesium Alloy by Hot Extrusion. Adv. Eng. Mater..

[8] Fakhar N., Sabbaghian M. (2021). A Good Combination of Ductility, Strength, and Corrosion Resistance of Fine-Grained ZK60 Magnesium Alloy Produced by Repeated Upsetting Process for Biodegradable Applications. J. Alloys Compd..

[9] Chen Y., Zhu Z., Zhou J. (2022). Study on the Strengthening Mechanism of Rare Earth Yttrium on Magnesium Alloys. Mater. Sci. Eng. A.

[10] Barezban M.H., Roumina R., Mirzadeh H., Mahmudi R. (2021). Effect of Gd on Dynamic Recrystallization Behavior of Magnesium During Hot Compression. Met. Mater. Int..

[11] Wang L., Zhang Z., Zhang H., Wang H., Shin K.S. (2020). The Dynamic Recrystallization and Mechanical Property Responses during Hot Screw Rolling on Pre-Aged ZM61 Magnesium Alloys. Mater. Sci. Eng. A.

[12] Wu J., Jin L., Dong J., Wang F., Dong S. (2020). The Texture and Its Optimization in Magnesium Alloy. J. Mater. Sci. Technol..

[13] Yu H., Liu Y., Liu Y., Wang D., Xu Y., Jiang B., Cheng W., Huang L., Tang W., Yu W. (2022). Enhanced Strength-Ductility Synergy in Mg-0.5 Wt%Ce Alloy by Hot Extrusion. Met. Mater. Int..

[14] Li R., Fu G., Xu Z., Su Y., Hao Y. (2018). Effect of Dynamically Recrystallized Grains on Rare Earth Texture in Magnesium Alloy Extruded at High Temperature. Adv. Eng. Mater..

[15] Li L., Wang Y., Zhang C., Wang T., Lv H. (2020). Effects of Yb Concentration on Recrystallization, Texture and Tensile Properties of Extruded ZK60 Magnesium Alloys. Mater. Sci. Eng. A.

[16] Wang W., Chen W., Jung J., Cui C., Li P., Yang J., Zhang W., Xiong R., Kim H.S. (2022). Asymmetry Evolutions in Microstructure and Strain Hardening Behavior between Tension and Compression for AZ31 Magnesium Alloy. Mater. Sci. Eng. A.

[17] Zhang D., Zhang D., Zhang Y., Chen S., Xu T., Meng J. (2021). Analysis of Strain Hardening Behavior in a Ductile Mg–Yb Based Alloy. Mater. Sci. Eng. A.

[18] Najafi S., Sabbaghian M., Sheikhani A., Nagy P., Fekete K., Gubicza J. (2022). Effect of Addition of Rare Earth Elements on the Microstructure, Texture, and Mechanical Properties of Extruded ZK60 Alloy. Met. Mater. Int..

[19] Zhou B., Zhu T., Jia H., Ma Z., Hao T., Wang J., Zeng X. (2023). Revealing the Weak Work-Hardening Behavior in Aged Mg–RE Alloys: A Synchrotron Radiation Diffraction Study. J. Alloys Compd..

[20] Shi Q., Wang C., Deng K., Fan Y., Nie K., Liang W. (2023). Work Hardening and Softening Behaviors of Mg-Zn-Gd-Ca Alloy Regulated by Bimodal Microstructure. J. Alloys Compd..

[21] Hou M., Deng K., Wang C., Nie K., Shi Q. (2022). The Work Hardening and Softening Behaviors of Mg–6Zn-1Gd-0.12Y Alloy Influenced by the VR/VD Ratio. Mater. Sci. Eng. A.

[22] Gubicza J. (2014). X-ray Line Profile Analysis in Materials Science.

[23] Ribárik G., Gubicza J., Ungár T. (2004). Correlation between Strength and Microstructure of Ball-Milled Al–Mg Alloys Determined by X-Ray Diffraction. Mater. Sci. Eng. A.

[24] Nagy P., Kaszás B., Csabai I., Hegedűs Z., Michler J., Pethö L., Gubicza J. (2022). Machine Learning-Based Characterization of the Nanostructure in a Combinatorial Co-Cr-Fe-Ni Compositionally Complex Alloy Film. Nanomaterials.

[25] Sabbaghian M., Fakhar N., Nagy P., Fekete K., Gubicza J. (2021). Investigation of Shear and Tensile Mechanical Properties of ZK60 Mg Alloy Sheet Processed by Rolling and Sheet Extrusion. Mater. Sci. Eng. A.

[26] Pang H., Li Q., Chen X., Chen P., Li X., Tan J. (2023). Dynamic Recrystallization Mechanism and Precipitation Behavior of Mg-6Gd-3Y-3Sm-0.5Zr Alloy During Hot Compression. Met. Mater. Int..

[27] Sabbaghian M., Mahmudi R., Shin K.S. (2021). Microstructural Evolution, Mechanical Properties, and Biodegradability of a Gd-Containing Mg-Zn Alloy. Metall. Mater. Trans. A.

[28] Lv S., Meng F., Lu X., Yang Q., Qiu X., Duan Q., Meng J. (2019). Influence of Nd Addition on Microstructures and Mechanical Properties of a Hot-Extruded Mg−6.0Zn−0.5Zr (Wt.%) Alloy. J. Alloys Compd..

[29] Gubicza J. (2017). Defect Structure and Properties of Nanomaterials.

[30] Liu T., Pan F., Zhang X. (2013). Effect of Sc Addition on the Work-Hardening Behavior of ZK60 Magnesium Alloy. Mater. Des..

[31] Liao H., Kim J., Liu T., Tang A., She J., Peng P., Pan F. (2019). Effects of Mn Addition on the Microstructures, Mechanical Properties and Work-Hardening of Mg-1Sn Alloy. Mater. Sci. Eng. A.

[32] Zhang D., Zhang D., Bu F., Li X., Li B., Yan T., Guan K., Yang Q., Liu X., Meng J. (2017). Excellent Ductility and Strong Work Hardening Effect of As-Cast Mg-Zn-Zr-Yb Alloy at Room Temperature. J. Alloys Compd..

